# Production of Monomeric Aromatic Compounds from Oil Palm Empty Fruit Bunch Fiber Lignin by Chemical and Enzymatic Methods

**DOI:** 10.1155/2015/891539

**Published:** 2015-12-22

**Authors:** Pei-Ling Tang, Osman Hassan, Mohamad Yusof Maskat, Khairiah Badri

**Affiliations:** ^1^School of Chemical Sciences and Food Technology, Faculty of Science and Technology, The National University of Malaysia (UKM), 43600 Bangi, Selangor, Malaysia; ^2^Polymer Research Center, Faculty of Science and Technology, The National University of Malaysia (UKM), 43600 Bangi, Selangor, Malaysia

## Abstract

In this study, oil palm empty fruit bunch (OPEFBF) was pretreated with alkali, and lignin was extracted for further degradation into lower molecular weight phenolic compounds using enzymes and chemical means. Efficiency of monomeric aromatic compounds production from OPEFBF lignin via chemical (nitrobenzene versus oxygen) and enzymatic [cutinase versus manganese peroxidase (MnP)] approaches was investigated. The effects of sodium hydroxide concentration (2, 5, and 10% wt.) and reaction time (30, 90, and 180 minutes) on the yield of aromatic compounds were studied. The results obtained indicated that nitrobenzene oxidation produced the highest yield (333.17 ± 49.44 ppm hydroxybenzoic acid, 5.67 ± 0.25 ppm* p*-hydroxybenzaldehyde, 25.57 ± 1.64 ppm vanillic acid, 168.68 ± 23.23 ppm vanillin, 75.44 ± 6.71 ppm syringic acid, 815.26 ± 41.77 ppm syringaldehyde, 15.21 ± 2.19 ppm* p*-coumaric acid, and 44.75 ± 3.40 ppm ferulic acid), among the tested methods. High sodium hydroxide concentration (10% wt.) was needed to promote efficient nitrobenzene oxidation. However, less severe oxidation condition was preferred to preserve the hydroxycinnamic acids (*p*-coumaric acid and ferulic acid). Cutinase-catalyzed hydrolysis was found to be more efficient than MnP-catalyzed oxidation in the production of aromatic compounds. By hydrolyzed 8% wt. of lignin with 0.625 mL cutinase g^−1^ lignin at pH 8 and 55°C for 24 hours, about 642.83 ± 14.45 ppm hydroxybenzoic acid, 70.19 ± 3.31 ppm syringaldehyde, 22.80 ± 1.04 ppm vanillin, 27.06 ± 1.20 ppm* p*-coumaric acid, and 50.19 ± 2.23 ppm ferulic acid were produced.

## 1. Introduction

Palm oil production is expanding to fulfill the growing worldwide needs for multiple uses of this commodity. However, the rapid growth of oil palm industry may have a negative impact on the environmental and social aspects. Indeed, sustainability of the supply chain of this commodity is a global challenge [1]. Nonetheless, the concept of biorefinery is an excellent alternative to ensure the sustainability of this commodity. In Malaysia, approximately 20 million tons of crude palm oil had been produced in 2014. With each ton of palm oil production, approximately a ton of empty fruit bunch fiber (OPEFBF) was generated. In order to achieve environmentally friendly production while maximizing profitability, OPEFBF must be utilized to generate maximum capacity of biorefinery, where each constituent of biomass (cellulose, hemicellulose, and lignin) must be recovered and converted into either food or nonfood fractions [2–4].

Pretreatment technology is a crucial step in lignocellulose biorefinery. A variety of pretreatment methods has been proposed with the aim to alter or remove the structural and compositional impediments to the hydrolysis of cellulose/hemicellulose. Delignification during pretreatment has been proven to improve enzymatic digestibility of biomass. However, pretreatment usually yields large quantity of black liquor that needs to be discharged at the end of the process [5, 6]. Direct disposal of black liquor is prohibited as it has a very high Chemical Oxygen Demand (COD) due to the presence of high content of lignin, phenolic compounds, silica, and so forth. Furthermore, lignin is a nonbiodegradable polymer [6–9]. Therefore, lignin recovery from the black liquor stream not only will assist the waste effluent management of the industry, but will also maximize the efficiency of biorefinery process.

Lignin is the most abundant amorphous aromatic polymer in lignocellulose. It is comprised of three main phenylpropane units, namely,* p*-hydroxyphenyl (H), guaiacyl (G), and syringyl (S). Future use of lignin depends on the possibilities of either degrading it into low molecular weight aromatics or as a multifunctional macromolecule. Lignin conversion into diverse value-added products played an important role in the economic viability of biorefinery. The methodologies of lignin conversion can be divided into three groups: (1) hydrothermal, (2) chemical, and (3) enzymatic [10, 11]. Hydrothermal treatment was reported as an excellent lignin conversion technique, where up to 50% of total lignin can be removed from the biomass. However, due to high temperature and pressure, condensation reaction among the highly reactive lignin fragments accelerates the generation of lignin-degraded products. In addition, the *β*-ether linkage of lignin was also cleaved rapidly, leads to monolignols and further its methyl ester formation [10]. The major group of lignolytic enzymes includes lignin peroxidases, manganese peroxidases, versatile peroxidases, and laccases. Due to the specific structures, different lignolytic enzyme has different mechanism in lignin degradation/oxidation and producing different compounds [12]. For chemical oxidation/degradation, it was mostly used in structural characterization of lignin polymer, based on the cleavage of *β*-aryl ether bonds. Until now, none of the chemical methods can be used commercially for the production of value-added compounds due to environmental concerns [11]. However, the advantage of chemical methods in terms of high yield production makes it superior over hydrothermal and enzymatic methods. Therefore, we aimed at the development of efficient and environmental benign chemical methods to cleave lignin efficiently under mild condition [10]. In spite of that, the discovery of lignolytic enzymes in white-rot fungi leads to the development of biotechnology in lignin conversion for small molecule chemicals production. Extracellular oxidative enzymes act synergistically and efficiently degrade lignin at mild condition [12]. Therefore, in this study, mild chemical treatments and enzymatic hydrolysis were designed to compare its effectiveness in the production of monomeric aromatic compounds under mild condition.

The types of product which are generated through lignin degradation/oxidation strongly depend on the applied methodologies and parameters [13]. Study [14–17] reported the production of monomeric aromatic aldehydes (such as vanillin,* p*-hydroxybenzaldehyde, and syringaldehyde) and hydroxycinnamic/phenolic acids (such as vanillic ac id, syringic acid, ferulic acid, and* p*-coumaric acid) through either chemical or enzymatic methods. All these aromatic compounds possess significant uses in food and pharmaceutical and nutraceutical applications [18–21].

Therefore, the main objective of this study was to effectively convert lignin fragment present in the black liquor after alkaline pretreatment of empty fruit bunch fiber (OPEFBF) into a series of novel monomeric aromatic compounds. The NaOH-lignin was initially recovered and then subjected to chemical (nitrobenzene and oxygen) and enzymatic (cutinase and MnP) oxidation/degradation. The effects of sodium hydroxide's concentration and time on the yield of monomeric aromatic compounds from chemical oxidation of lignin were investigated. Besides, reaction condition of cutinase in hydrolyzing NaOH-lignin was optimized for pH, temperature, lignin concentration, enzyme concentration, and time. The performance of each method was evaluated based on the types and yields of the monomeric aromatic compounds produced.

## 2. Materials and Methodology

### 2.1. Materials

The oil palm empty fruit bunch fiber (OPEFBF) provided by Seri Ulu Langat Sdn. Bhd., Dengkil, Selangor, Malaysia, was used as the substrate for this study. The fresh OPEFBF was washed with tap water and sun-dried until the moisture content reached approximately 10-11%. The dried OPEFBF was cut into the size of 5–8 cm and kept in sealed plastic bag at ~4°C until further usage.

Chemicals used included sodium hydroxide (NaOH), nitrobenzene, chloroform, ethyl acetate, HPLC grade methanol, dimethyl sulfoxide (DMSO),* p*-nitrophenyl butyrate (PNPB), hydrogen peroxide, manganese sulphate, disodium succinate, disodium malate, 2,2-Azino-bis(3-ethylbenzthiazoline-6-sulfonic acid) (ABTS) diammonium salt (Merck, Germany), dipotassium hydrogen phosphate, potassium dihydrogen phosphate, sodium phosphate, and sodium chloride (Sigma Aldrich, USA). Phenolic compounds used as a reference standard were hydroxybenzoic acid,* p*-hydroxybenzaldehyde, vanillic acid, vanillin, syringic acid, syringaldehyde,* p*-coumaric acid, and ferulic acid (Sigma Aldrich, USA). All chemicals used were of analytical grade.

Enzymes used in this study were Novozym 51032 (Novozymes, Denmark) and manganese peroxidase (Sigma Aldrich, USA). The activities of Novozym 51032 (*Fusarium solani pisi*) and manganese peroxidase (MnP) (*Phanerochaete chrysosporium*) were determined according to the modified protocol proposed by [22] and [23], respectively. The reaction mixtures contained 10 *µ*L 50 mM PNPB solution and 100 *µ*L Novozym 51032, in a final volume of 1 mL of 0.1 M sodium phosphate containing 0.9% sodium chloride at pH 7.2, was prepared. The enzyme activity of Novozym 51032 was determined by monitoring the increase of absorbance at 400 nm, with no-enzyme mixture as a blank. For MnP activity determination, 20 *µ*L MnP was added to mixture containing 1 mM manganese sulphate and 72 *µ*M ABTS diammonium salt in 1 mL of 50 mM succinate-10 Mm malate buffer at pH 4.5. About 2.5 *µ*L of 20 mM hydrogen peroxide was added to the mixture to initiate the reaction. The activity of MnP was determined by monitoring the increased absorbance at 415 nm as a function of time. The protein concentration of both enzymes was determined through Bradford method by using Bio-Rad Quick Start Bradford assay kit (Bio-Rad, USA). Bovine serum albumin (BSA) was used as the reference standard. About 1 mL of Bio-Rad 1x reagent was mixed with 20 *µ*L of BSA at a series of concentrations/enzyme solutions. The absorbance of the mixtures was read at wavelength 595 nm by using VersaMax ELISA microplate reader and analysed with SoftMax Pro software (Molecular Device, USA). Specific activity of Novozym 51032 and MnP was found at about 673.1 U mg^−1^ and 31.3 U g^−1^, respectively.

### 2.2. Methodology

#### 2.2.1. Lignin Preparation

OPEFBF delignification was conducted by soaking the OPEFBF in 50 g L^−1^ NaOH solution at a solid-to-liquid ratio of 1 : 10. The treatment was conducted at 120°C in an autoclave for 120 minutes. The reaction was conducted in a capped 500 mL Schott bottle. After treatment, the treated OPEFBF was separated from the black liquor by vacuum filtration using muslin cloth. pH of the recovered black liquor was adjusted to pH 2 using concentrated sulphuric acid. Lignin precipitate was separated from the liquor by centrifugation at 10000 rpm for 10 minutes (Eppendorf 5804 centrifuge, Hamburg, Germany). The recovered lignin fragment was washed with distilled water and oven-dried at 105°C overnight. The dried lignin fragment was crushed and ground into fine powder using mortar and pestle [24, 25]. The lignin powder was kept in a tightly capped bottle at room temperature until used for experiments.

#### 2.2.2. Nitrobenzene Oxidation

Nitrobenzene oxidation of lignin was conducted with 2% wt. lignin in 10 mL of 2, 5, and 10% wt. NaOH solutions. About 0.5 mL nitrobenzene was added to the lignin solution, while mixture without nitrobenzene was fixed as experimental control. The oxidation reaction was carried out in an autoclave at 130°C for 30, 90, and 180 minutes. After oxidation, 30 mL chloroform was added to the mixture to remove excess nitrobenzene and its reduction products. Chloroform layer was then discharged by centrifugation at 3000 rpm for 3 minutes. The oxidation products extraction was initiated by adding concentrated sulphuric acid to the reaction mixture (lower layer) to achieve pH 2. The extraction was conducted thrice with an equal volume of ethyl acetate. The ethyl acetate was then evaporated in a rotary evaporator. The dried oxidation products were resolubilized in methanol and subjected to HPLC analysis [26].

#### 2.2.3. Direct Oxygen Oxidation

For direct oxygen oxidation, 2% wt. lignin was dissolved in 10 mL 2, 5, and 10% wt. NaOH solutions separately. The reaction was conducted in a 100 mL 2-neck round-bottom flask. A condenser was connected to the flask and oxygen gas was bubbled into the lignin solution continuously throughout the entire reaction period. The reaction mixture was heated to 100°C and maintained for 30, 90, and 180 minutes. Oxygen oxidation was carried out under atmospheric pressure [27]. Experiment control was conducted as the prescribed procedure, where oxygen gas was not channeled into the reaction mixture. Once the reaction was completed, heating was stopped and the mixture was allowed to cool, before the pH was adjusted to pH 2 by adding concentrated sulphuric acid. Extraction of the oxidation products was conducted as the prescribed protocol in Section 2.2.2 by using ethyl acetate.

#### 2.2.4. Enzyme Hydrolysis Using Cutinase (Novozym 51032)

Due to the insolubility of lignin in aqueous buffer solution, lignin dissolution was conducted prior to the enzymatic hydrolysis. Initially, 0.2 g lignin was dissolved in 1 mL DMSO. To study the effect of pH on the enzyme catalytic activity, about 10 mL of 25 mM phosphate buffer at pH 7, 8, 10, and 12 was added to the solution. Lignin hydrolysis by Novozym 51032 was carried out with 2.5 mL cutinase g^−1^ lignin at 55°C with 150 rpm agitation for 24 hours [28]. Once the catalysis was completed, the mixture was heated in boiling water for 5 minutes to inactivate the enzyme. pH of the hydrolysate was then adjusted to pH 5 with 10% wt. hydrochloric acid. The lignin fragment was separated from the hydrolysate by centrifugation at 3000 rpm for 3 minutes. Aromatic compounds present in the hydrolysate were analyzed using HPLC. Stepwise optimization of the lignin concentration (2, 5, 8, and 10% wt.), enzyme concentration (0.625, 170 1.25, 1.875, and 2.5 mL cutinase g^−1^ lignin), time (24, 48, and 72 hours), and temperature (40, 55, and 65°C) on the efficiency of enzymatic catalysis were carried out.

#### 2.2.5. Manganese Peroxidase (MnP) Oxidation

Enzymatic oxidation of lignin by MnP was conducted according to the modified procedure suggested in [23]. A reaction mixture comprised of 8% wt. lignin, 1 mM manganese sulphate, and 0.625 mL MnP g^−1^ lignin in 50 mM succinate-10 mM malate buffer pH 4.5 was prepared. The enzymatic oxidation reaction was conducted at 30°C with 200 rpm agitation for 48 hours. The reaction was initiated by the addition of 20 mM hydrogen peroxide. Once the oxidation was completed, enzyme inactivation was carried out by heating the reaction mixture to 80°C for 5 minutes. The lignin fragment was then separated from the liquor by centrifugation at 3000 rpm for 3 minutes. The liquor and the recovered lignin were subjected to HPLC and FTIR analysis, respectively.

#### 2.2.6. High Performance Liquid Chromatography (HPLC) Analysis

The monomeric aromatic compounds identification and quantification were performed by HPLC equipped with an ultraviolet detector at wavelength 280 nm (Waters, Milford, USA). A 250 × 4.6 mm HPLC column Gemini C18 (Phenomenex, California, USA) with 5 *µ*m particle size was used, and the sample was eluted using 0.1% acetic acid (solvent A) and methanol (solvent B). The mobile phase gradient was fixed at 80 : 20 (A : B) from 0 to 24 min, 60 : 40 (A : B) from 24 to 27 min, 20 : 80 (A : B) from 27 to 36 min, and 80 : 20 (A : B) from 36 to 40 min. The mobile phase was filtered through a 47 mm nylon membrane with a pore size of 0.45 *µ*m and degassed. The compounds separation was conducted at a constant flow rate of 1 mL min and 10 *µ*L injection volume. Aromatic compounds identification was carried out by using reference standards comprising of hydroxybenzoic acid,* p*-hydroxybenzaldehyde, vanillic acid, vanillin, syringic acid, syringaldehyde,* p*-coumaric acid, and ferulic acid. Caffeine was used as an internal standard for quantification. All of the standards and samples were filtered through a 0.22 *µ*m nylon membrane prior to analysis. Data acquisition was performed with Breeze software (Waters, Milford, USA) [24].

## 3. Results and Discussion

### 3.1. Lignin Oxidation by Nitrobenzene

**Figure 1:**
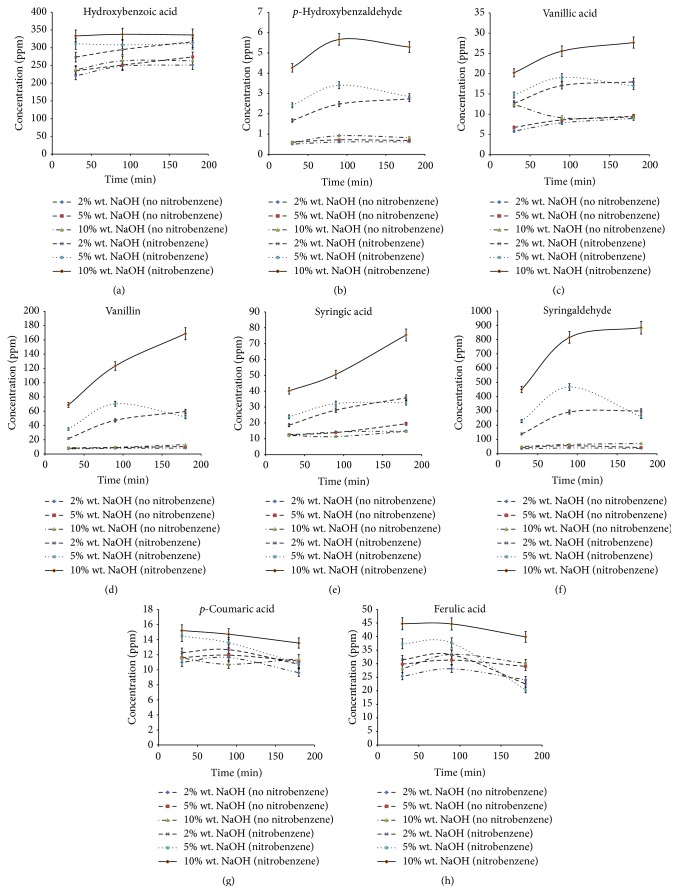
Effects of sodium hydroxide's concentration and time on the yields of (a) hydroxybenzoic acid, (b)* p*-hydroxybenzaldehyde, (c) vanillic acid, (d) vanillin, (e) syringic acid, (f) syringaldehyde, (g)* p*-coumaric acid, and (h) ferulic acid.

Figure 1 demonstrated the effects of sodium hydroxide concentration and time on the yield of aromatic compounds from lignin nitrobenzene oxidation. Based on the results, it was found that the products generated from lignin nitrobenzene oxidation included hydroxybenzoic acid,* p*-hydroxybenzaldehyde, vanillic acid, vanillin, syringic acid, syringaldehyde,* p*-coumaric acid, and ferulic acid. These eight compounds also have been identified in the study of [29, 30] after nitrobenzene oxidation of OPEFBF lignin. According to Figure 1, the yield of all oxidation products in the nitrobenzene-contained medium was higher than those in the absence. This result indicated that nitrobenzene had significantly improved the oxidation reaction, and thus the yield of all oxidation products. Large increment was observed, specifically in the yields of* p*-hydroxybenzaldehyde, vanillic acid, vanillin, syringic acid, and syringaldehyde. Besides, the results of Figure 1 also demonstrated that the yields of all oxidation products, except* p*-coumaric acid and ferulic acid, were increased, followed by the increased time. The yields of* p*-coumaric acid and ferulic acid were found to be decreased when oxidation reaction prolonged. In spite of that, the increase of NaOH concentration also was found to be increase the yield of all oxidation products. However, in the absence of nitrobenzene, the effect of NaOH concentration on the yield of oxidation products was not apparent. Based on the results obtained, the highest yield of all oxidation products was attained at 10% wt. NaOH solution but at different optimum time. The yields of hydroxybenzoic acid (333.17 ± 49.44 ppm with the recovery of 16.66 ± 2.47 mg g^−1^ lignin),* p*-coumaric acid (15.21 ± 2.19 ppm with the recovery of 0.76 ± 0.11 mg g^−1^ lignin), and ferulic acid (44.75 ± 3.40 ppm with the recovery of 2.24 ± 0.17 mg g^−1^ lignin) were achieved maximum after 30 minutes of reaction, while the maximum yield of* p*-hydroxybenzaldehyde (5.67 ± 0.25 ppm with the recovery of 0.28 ± 0.01 mg g^−1^ lignin), vanillic acid (25.57 ± 1.64 ppm with the recovery of 1.28 ± 0.08 mg g^−1^ lignin), and syringaldehyde (815.26 ± 41.77 ppm with the recovery of 40.76 ± 2.09 mg g^−1^ lignin) was achieved at 120 minutes reaction. The longest reaction time (180 min) needed to achieve the maximum yield was of syringic acid (75.44 ± 6.71 ppm with the recovery of 3.77 ± 0.34 mg g^−1^ lignin) and vanillin (168.68 ± 23.23 ppm with the recovery of 8.43 ± 1.16 mg g^−1^ lignin). No further yield improvement was observed on hydroxybenzoic acid, when the oxidation time was extended. Nevertheless, the extended oxidation reaction up to 180 min caused the yield of* p*-coumaric acid and ferulic acid diminished. However, the yields of* p*-hydroxybenzaldehyde, vanillic acid, and syringaldehyde were found to be constant, and even the oxidation had been extended to 180 min.

Among these compounds,* p*-hydroxybenzaldehyde, vanillin, and syringaldehyde were the oxidized products of* p*-hydroxyphenyl (H), guaiacyl (G), and syringyl (S) monomer unit of lignin, respectively [30]. According to the results obtained, syringaldehyde was the sole product of OPEFBF lignin nitrobenzene oxidation, followed by hydroxybenzoic acid and vanillin. Under the commonly used temperature setting of nitrobenzene oxidation (~170°C), vanillin and syringaldehyde were always reported to be the main products, to indicate the ratio of guaiacyl and syringyl units in the lignin [27]. The work in [25, 31] reported that OPEFBF lignin contained the highest proportion of noncondensed syringyl units with small amount of noncondensed guaiacyl units and fewer* p*-hydroxyphenyl units. Therefore, the highest yield of syringaldehyde was originated from the oxidation of the majority syringyl units of OPEFBF lignin. However, hydroxybenzoic acid was found to be the second abundant product, rather than the expected vanillin. The work in [25] revealed that OPEFBF lignin contained a significant amount of esterified* p*-hydroxybenzoic acid, while the conversion of* p*-coumaric acid into* p*-hydroxybenzaldehyde and hydroxybenzoic acid was also suggested by [26]. Therefore, high yield of hydroxybenzoic acid was reasonable in this study. During nitrobenzene oxidation at low temperature, the cleavages of ester and some ether linkages in the lignin network are predominant. Thus, the release of hydroxycinnamic acid from lignin was promoted. Besides, the lignin was firstly undergoing based-catalyzed hydrolysis to produce large fraction of benzylic hydroxyl groups which is soluble in the alkaline medium. The benzylic hydroxyl groups were then oxidized and cleaved at the C_*α*_-C_*β*_ bond to form benzaldehyde and its derivatives. Therefore, in the medium of strong alkalinity and the presence of nitrobenzene as oxidant, free* p*-coumaric acid tends to convert into* p*-hydroxybenzaldehyde and then hydroxybenzoic acid [26]. This also explained the observation of the yield increment of* p*-hydroxybenzaldehyde and hydroxybenzoic acid along with the* p*-coumaric acid reduction during the oxidation reaction.

Under alkaline condition, ester linkages joining phenolic acid are easily cleaved, even at room temperature. However, phenolic benzyl aryl ethers only cleaved at high temperature and with the presence of oxidants [32]. Therefore, with the presence of nitrobenzene, the yield of syringaldehyde which is formed from the oxidative cleavage of syringyl unit increased dramatically, especially under strong alkaline medium. Large increment of* p*-hydroxybenzaldehyde and vanillin was mainly due to the conversion of* p*-coumaric acid and ferulic acid, respectively. Nonetheless, drastic increment of vanillin yield also could be due to the oxidative cleavage of guaiacyl units in the lignin network. In addition, alkalinity also had been proven to play a significant role during nitrobenzene oxidation. Alkaline nitrobenzene oxidation induced alkaline hydrolysis of interunit ether bonds to depolymerize and solubilize lignin. Once the aldehyde groups are formed, base-catalyzed hydrolysis of ether bonds can occur at higher rate, particularly at high NaOH concentration [26]. The rate of oxidative cleavage of lignin into aromatic aldehyde was determined by the rate of retroaldol cleavage of C_*α*_-C_*β*_ bond of the phenylpropane units. High concentration of hydroxide ion in the presence of oxidant has been proven to accelerate the rate of retroaldol cleavage. Mechanism of nitrobenzene oxidation of lignin was proposed by [33, 34].

According to [17], prolonged reaction time accelerated the oxidation of* p*-coumaric acid and ferulic acid into their corresponding aldehydes and then its carboxylic acids by Cannizzaro reaction. Therefore, extended oxidation time was associated with the increase of* p*-hydroxybenzaldehyde, vanillin, hydroxybenzoic acid, and vanillic acid yields. This finding was in accordance with the results shown by the study of [35, 36]. Besides, increased oxidation time also suggested the promotion of oxidative depolymerization of some specific phenylpropane monomeric units in the lignin network, which is unable to be solubilized at the early stage of reaction [17]. Thus, at longer oxidation time, the aromatic aldehyde/carboxylic acid yields no longer primarily originated from the oxidation of the hydroxycinnamic acids released at the early stage of reaction, but they were also formed from the oxidative cleavage of phenylpropane units of lignin.

Different optimum time for optimum yield of different oxidation products indicated that oxidation time has direct influence on the products formed during nitrobenzene oxidation. Different molecular structures of different molecules give rise to different susceptibilities to oxidative degradation during nitrobenzene oxidation in the formation of aromatic compounds [37]. The reaction time affected the severity of nitrobenzene oxidation. Thus, by extending the reaction time, esterified hydroxycinnamic acids were initially released, followed by the formation of aromatic aldehydes through oxidative cleavage of lignin network and oxidation of hydroxycinnamic acids and finally the conversion of aldehydes into its corresponding carboxylic acids. This finding was in agreement with the study [17].

### 3.2. Direct Oxygen Oxidation of Lignin

**Figure 2:**
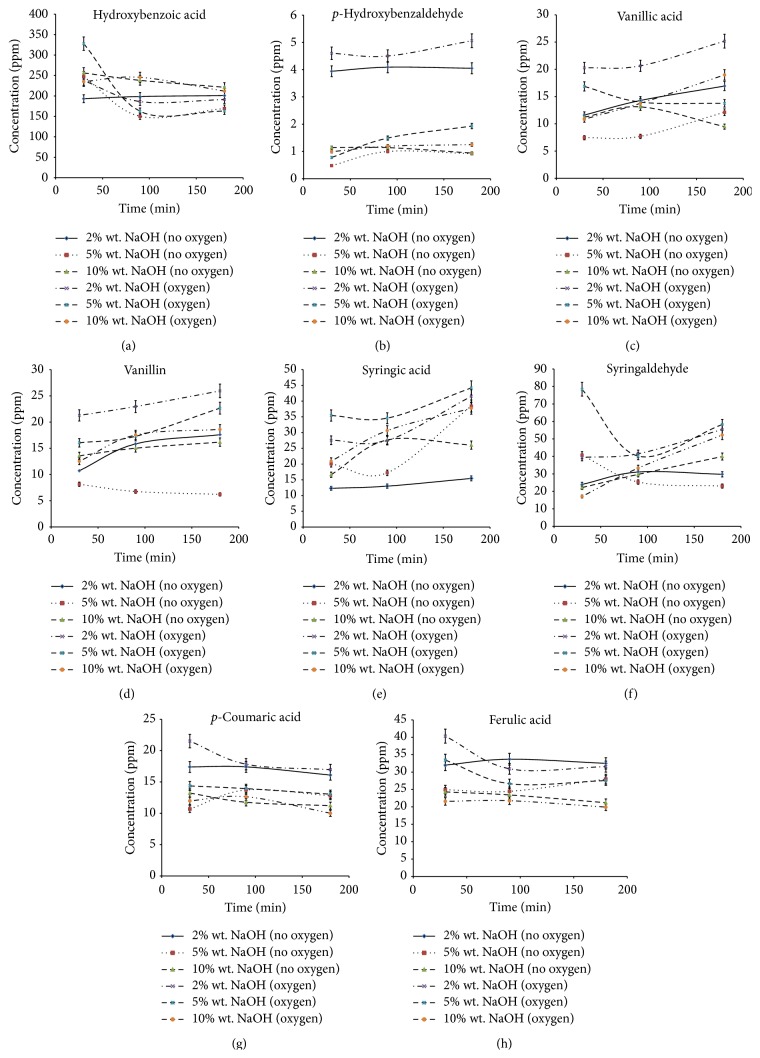
Effects of sodium hydroxide's concentration and time on the yields of (a) hydroxybenzoic acid, (b)* p*-hydroxybenzaldehyde, (c) vanillic acid, (d) vanillin, (e) syringic acid, (f) syringaldehyde, (g)* p*-coumaric acid, and (h) ferulic acid during oxygen oxidation at 100°C.

Figure 2 showed the effects of sodium hydroxide's concentration and time on the changes of the yield of oxidation products during lignin oxygen oxidation. The identified oxidation products after oxygen oxidation were similar to those detected in the liquor from nitrobenzene oxidation, but at a lower yield. Based on the results obtained, hydroxybenzoic acid was identified as the main product in this reaction. The yield of hydroxybenzoic acid after lignin oxygen oxidation was the highest (in the range of 150–330 ppm) among the formed oxidation products, regardless of the oxidation condition. In addition, the presence of oxygen was found to significantly improve the yield of all oxidation products, particularly at lower NaOH concentration. The data from the experiment also revealed that the yields of* p*-hydroxybenzaldehyde,* p*-coumaric acid, and ferulic acid in the reaction medium of 10% wt. NaOH were not significant (*p* > 0.05), neither was the presence of oxygen. Therefore, such finding suggested that the effect of oxygen in oxidation is more significant at low NaOH concentration. In general, based on the results obtained, low yield of oxidation products was found in reaction with high NaOH concentrations. On the other hand, the yields of oxidation products had improved with the extension of reaction time, except for hydroxybenzoic acid,* p*-coumaric acid, and ferulic acid.

For maximum recoveries of all potential aromatic compounds, direct oxygen oxidation was conducted at 2% wt. NaOH for 180 minutes to produce maximum yield of* p*-hydroxybenzaldehyde (5.06 ± 0.05 ppm with the recovery of 0.25 ± 0.01 mg g^−1^ lignin), vanillic acid (25.17 ± 1.16 ppm with the recovery of 1.26 ± 0.06 mg g^−1^ lignin), vanillin (25.94 ± 2.25 ppm with the recovery of 1.30 ± 0.11 mg g^−1^ lignin), and syringic acid (41.52 ± 0.64 ppm with the recovery of 2.08 ± 0.03 mg g^−1^ lignin). However, only the shortest reaction time (30 min) was required for the optimum production of* p*-coumaric acid (21.53 ± 1.10 ppm with the recovery of 1.08 ± 0.06 mg g^−1^ lignin) and ferulic acid (40.33 ± 2.59 ppm with the recovery of 2.02 ± 0.13 mg g^−1^ lignin) at 2% wt. NaOH, while hydroxybenzoic acid (328.02 ± 39.55 ppm with the recovery of 16.40 ± 1.98 mg g^−1^ lignin) and syringaldehyde (78.43 ± 0.88 ppm with the recovery of 3.92 ± 0.04 mg g^−1^ lignin) at 5% wt. NaOH. The yields of these oxidation products were either reduced or kept constant, if the oxidation time was extended up to 180 min. This finding suggested the interactive effect of NaOH concentration and reaction time in getting the optimum yield of each compound. Different optimum reaction conditions were identified for the production of distinct compound. Study [14] proposed that such occurrence was mainly due to different stability and susceptibility of each compound to different oxidation conditions.

The highest yield of hydroxybenzoic acid after oxygen oxidation was believed to be due to the release of esterified hydroxybenzoic acid from the lignin network. Under mild oxidation environment, as designed in this experiment, the cleavages of ester and some ether bonds in the lignin network became predominant. The rate of oxidative cleavage of lignin phenylpropane units was minimal under mild reaction condition. Therefore, syringaldehyde and vanillin, which are the common aromatic aldehydes of lignin oxidation, did not appear as the major products in this experiment [25, 26].

In addition, discovery of high rate oxygen oxidation at low NaOH concentration in this study has been found to be contradicting with the results from the previous study. According to [16], the efficiency of oxygen oxidation reaction depends on the oxygen pressure, sodium hydroxide concentration, and temperature. Among these factors, oxygen exerted the most intense effect on the reaction efficiency, particularly at high NaOH concentration. This contradiction was believed to be due to the effect of reaction temperature. Under the same reaction condition, the trend of changes in products yields of nitrobenzene oxidation at 130°C and oxygen oxidation at 100°C was found to change reciprocally with the increase of NaOH concentrations. At temperature 130°C, the products yield was increased at high NaOH concentration. Therefore, the combined effect of NaOH concentration and temperature was suspected to be the key factor of this finding. Oxygen oxidation is a multiphase gas-liquid-solid system. Indeed, the rate of the mass transfer from gas phase to liquid affected the efficiency of the system. Lignin oxidation by oxygen was reported to follow the mechanism of radical chain reaction proposed by [38, 39]. High temperature and alkali concentration were recommended in order to achieve high rate of initiation step (reaction of phenolate anion with oxygen molecule) during oxygen oxidation. However, the results of study by [38] showed that the initiate rate of oxygen consumption reduced from 0.12 to 0.08 g min^−1^ by the increase of NaOH concentration from 80 to 120 g L^−1^ at 160°C. Therefore, oxidation at lower temperature might further reduce the rate of oxygen absorption in the reaction at high NaOH concentration. Low oxygen absorption in the medium will reduce the rate of initiation and thus the yields of oxidation products. Nevertheless, verification on this hypothesis is needed in further study.

Furthermore, subsequent oxidation of* p*-coumaric acid and ferulic acid at prolonged reaction time probably was the main cause for the decrease of these compounds [14, 17]. In addition, the data also indicated that the maximum yield of these compounds was achieved at the shortest reaction time (30 min). Thus, the extended reaction time (>30 min) caused oxygen to loose its role in improving the yield of these compounds. contrarily, it accelerates the rate of subsequent oxidation of* p*-coumaric acid and ferulic acid in the formation of aldehydes and then carboxylic acid [17]. Moreover, such finding suggested that, at longer reaction time, atmospheric oxygen is enough to fulfill the oxygen needs for mild oxygen oxidation in releasing the esterified hydroxycinnamic acid from the lignin network. In this study, temperature was suspected to be the primary limiting factor for oxidation by direct oxygen reaction. Study [38] demonstrated the effect of thermodynamic in the mechanism of lignin oxidation.

### 3.3. Lignin Hydrolysis by Cutinase Enzyme (Novozym 51032)

Cutinase is a serine esterase (cutin hydrolase), particularly acting on the carboxylic ester bonds. Due to the fact that its active site cleft is partly covered by 2 disulfide bridges formed by amino acid chains (hydrophilic nature) and not buried under surface loop (hydrophobic nature), interfacial activation is unnecessary for its catalysis, as it is easily accessible to solvent and substrate. Moreover, oxyanion hole in cutinase structure also allow catalytic activities towards a broad variety of esters, from soluble to insoluble types [40]. Until now, catalysis of cutinase toward lignin was not reported in any publication. In this study the hypothesis was initially made by expecting the cutinase to cleave ester bonds on the lignin substrate. Ester bonds cleavage in lignin network was hypothesized to generate esterified phenolic compounds and modify the lignin molecular structure.  Table 1 presented the effects of pH, lignin concentration, enzyme concentration, time, and temperature on the catalytic efficacy of enzyme cutinase Novozym 51032 on lignin hydrolysis. The aromatic compounds detected at a considerable concentration were hydroxybenzoic acid, syringaldehyde, vanillin,* p*-coumaric acid, and ferulic acid. The highest yield was achieved by hydroxybenzoic acid (642.83 ± 14.45 ppm), followed by syringaldehyde (70.19 ± 3.31 ppm) and ferulic acid (50.19 ± 2.23 ppm) and then* p*-coumaric acid (27.06 ± 1.20 ppm) and eventually vanillin (22.80 ± 1.04 ppm). These optimum yields were achieved by hydrolyzed lignin with cutinase at pH 8 and 55°C for 24 hours with 8% wt. lignin and 0.625 mL cutinase g^−1^ lignin. Under these reaction conditions, about 8.04 ± 0.18 mg hydroxybenzoic acid, 0.88 ± 0.04 mg syringaldehyde, 0.63 ± 0.02 mg ferulic acid, 0.34 ± 0.02 mg* p*-coumaric acid, and 0.29 ± 0.01 mg vanillin have been successfully recovered from each gram of lignin through this catalysis.

It was known that hydroxybenzoic acid,* p*-coumaric acid, and ferulic acid were esterified components in the lignin network. Thus, the finding suggested that the cleavage of ester bonds within the lignin network by cutinase was the leading factor in the production of these compounds. Because cutinase is not an oxidative enzyme, syringaldehyde might emerge from syringyl alcohol units, which was released after lignin network deconstruction by the action of cutinase. Low yield of vanillin was most likely formed by the oxidation of the released free ferulic acid. Slow rate oxidation may happen under atmospheric oxygen condition. Nonetheless, further study on the mechanism of cutinase toward lignin is necessary for clear and definite understanding in this catalysis.

Based on the results shown in Table 1(a), pH 8 was proven to be the optimum pH for maximum performance of cutinase in lignin hydrolysis. At this pH, hydrolysate with the maximum content of hydroxybenzoic acid (165.30 ± 2.51 ppm), syringaldehyde (14.94 ± 0.36 ppm), vanillin (8.02 ± 0.32 ppm),* p*-coumaric acid (7.72 ± 0.20 ppm), and ferulic acid (14.06 ± 0.13 ppm) were produced. Besides, the results also clearly indicated that the products yield and recovery increased with the increase of pH to an optimum. After that, increase in pH will be accompanied by decrease in yield. Increase of pH from 7 to 10 had improved the products yield and recovery significantly (*p* < 0.05). Increase to pH 12 had caused adverse effects on both yield and recovery. This result was in accordance with the study of [41]. Cutinase catalysis rate was reported to be very low at pH below 7. The reaction rate increased rapidly as pH was increased from 7.5 to 10. The finding showed that the action of cutinase on lignin will produce aromatic compounds. Its maximum yield was achieved at the optimum pH of enzyme. However, maximum yield of vanillin was achieved at pH 12. Study [34] indicated that the retroaldol reaction played significant role in the alkaline oxidative destruction of lignin in vanillin production. High alkalinity has been reported to increase the rate of retro-aldol reaction in the formation aldehyde. In the reaction of lignin oxidation, phenolate anion formed following the dehydration of lignin structural units. Quinone methide radical formed due to the abstraction of an electron from the phenolate anion. Acid dissociation of quinone methide radical gives rise to the formation of radical anion with a partial delocalization of the unpaired electron in the *γ*-position of the propane chain. Eventually, vanillin is formed as a result of oxidation reaction in the *γ*-position, followed by retroaldol reaction of substituted coniferyl aldehyde [34]. In addition, ferulic acid was slowly converted to vanillin, under highly alkaline condition [36].

In addition, the results in Table 1(b) also showed that the highest products' yields were achieved at the highest lignin's concentration (10% wt.). About 733.19 ± 26.68 ppm of hydroxybenzoic acid, 77.78 ± 3.78 ppm of syringaldehyde, 25.39 ± 0.78 ppm of vanillin, 29.13 ± 2.72 ppm of* p*-coumaric acid, and 56.95 ± 2.52 ppm of ferulic acid were successfully produced at this condition. However, high substrate content was associated with lower compounds recovery. The products recoveries were found to be reduced, while the substrate content increased from 2% wt. to 10% wt. According to [41], the rate of enzymatic hydrolysis increased by the increase in substrate concentration. Further increase of substrate concentration will cause slight decrease in the rate of catalysis. The decrease could be due the absorption of cutinase onto the insoluble substrate. High concentration of substrate in the reaction mixture may promote high level of nonproductive enzyme absorption onto the substrate. In addition, the large molecular weight and compact molecular structure of lignin might also contribute to low substrate binding at the active site of cutinase. Study [42] proposed that enzyme activity toward large molecular weight substrate could be enhanced by substituting the residues near the active site with a smaller residue to create more space in the active site. Therefore, high substrate level must be avoided in the native cutinase catalysis, particularly toward high molecular weight substrate. According to the data obtained, optimum recoveries of most of the compounds were achieved at 5% wt. lignin, except for vanillin. The recoveries of these compounds were increased by increasing the lignin's concentration from 2 to 5% wt., but they declined when the concentration was increased toward 10% wt. This finding was the same as study [41]. Ultimately, 8% wt. lignin was selected as the optimum point for the maximum catalytic performance of cutinase, to obtain hydrolysate with the highest amount of aromatic compounds.

Furthermore, the results in Tables 1(c) and 1(d) showed that the increase in enzyme concentration and reaction time produced lower yields and recoveries of aromatic compounds from lignin hydrolysis by cutinase, except vanillin. By increased enzyme concentration from 0.625 to 2.5 mL cutinase g^−1^ lignin, the yields of hydroxybenzoic acid, syringaldehyde,* p*-coumaric acid, and ferulic acid were diminished from 642.83 ± 14.45 ppm, 70.19 ± 3.31 ppm, 27.06 ± 1.20 ppm, and 50.19 ± 1.23 ppm to 542.94 ± 27.88 ppm, 54.94 ± 4.67 ppm, 19.69 ± 0.25 ppm, and 46.93 ± 2.81 ppm, respectively. However, vanillin yield was not significantly affected by the increase of enzyme concentration. Vanillin yields from hydrolysis with 0.625 and 2.5 mL cutinase g^−1^ lignin were 22.80 ± 1.04 ppm and 20.77 ± 1.12 ppm, respectively. Moreover, the results also demonstrated that the extended reaction time from 24 to 72 hours had reduced the yield of syringaldehyde (from 70.19 ± 3.31 to 34.03 ± 2.01 ppm),* p*-coumaric acid (from 27.06 ± 1.20 to 23.76 ± 1.63 ppm), and ferulic acid (from 50.19 ± 2.23 to 43.39 ± 1.10 ppm), while it increased the yield of vanillin (from 22.80 ± 1.04 to 32.64 ± 2.35 ppm). Nevertheless, the yield hydroxybenzoic acid was unaffected, even at the prolonged reaction time. There was 642.83 ± 14.45 and 649.62 ± 21.35 ppm of hydroxybenzoic acid produced at 24 and 72 hours reaction, respectively. According to study [43], the increase of both of these factors has resulted in an increase of cotton wax removal from cotton fabric. Once the optimum point was reached, no additional removal was observed by further increase in enzyme concentration and reaction time. However, opposite results have been demonstrated in this study. The mechanism of action of cutinase toward lignin is still not fully understood. Thus, it was unclear why slight reduction of yield aromatic compounds was observed when these factors were increased. In spite of that, the lowest enzyme's loading (0.625 mL cutinase g^−1^ lignin) and shortest reaction time (24 hours) were found to be adequate in achieving maximum recovery of aromatic compounds from lignin hydrolysis. High vanillin yield was probably due to the conversion of free ferulic acid after a prolonged reaction time. This suggestion was supported with the results as shown, where the decrease of ferulic acid correlates very well with the increase of vanillin.

Temperature is the most critical factor affecting the performance and stability of enzyme catalysis process. Based on the results obtained, it was distinctly indicated that 55°C was the optimum temperature for cutinase catalysis. At this optimum temperature, about 557.80 ± 12.76 ppm of hydroxybenzoic acid, 53.94 ± 1.20 ppm of syringaldehyde, 21.68 ± 0.50 ppm of vanillin, 22.81 ± 1.44 ppm of* p*-coumaric acid, and 46.94 ± 1.59 ppm of ferulic acid were produced. The release of aromatic compounds during hydrolysis was in similar manner to the effect of pH. The yields of compounds and recoveries were lower when the hydrolysis was performed at the lowest (40°C) and highest (65°C) temperature. According to a study [42], cutinase has a good stability in the temperature range of 20–50°C. Study [44] demonstrated that optimum activity of cutinase was achieved at temperature range of 40–50°C. Therefore, the optimum temperature determined in this study has been supported by previous investigators. However, the maximum vanillin (23.44 ± 2.21 ppm; 0.29 ± 0.03 mg g^−1^ lignin) and syringaldehyde (60.12 ± 4.26 ppm; 0.75 ± 0.05 mg g^−1^ lignin) yield and recovery were achieved at the highest temperature (65°C). High yield of both compounds can be attributed to the release of appropriate phenolics from lignin after the cutinase reaction, then followed by slow oxidation under high temperature (65°C) and alkalinity (pH 8).

### 3.4. Lignin Oxidation by Manganese Peroxidase (MnP)

Manganese peroxidase (MnP) is a class II peroxidase group in* basidiomycetous* fungi. It is an extracellular ligninolytic enzyme containing a heme protein and a highly specific Mn^2+^ binding site. During MnP-catalyzed oxidation, the chelated Mn^3+^ ion, but not MnP itself, acts as a low molecular weight mediator and freely diffusible oxidizing species which will ultimately perform oxidative depolymerization of lignin/lignin model compounds [13, 45, 46].  Table 2 showed the yields and recoveries of phenolic compounds from manganese peroxidase (MnP) catalysis on lignin. The results clearly indicated that hydroxybenzoic acid, vanillic acid, vanillin,* p*-coumaric acid, and ferulic acid were generated at a pronounced level under the designed reaction condition. About 459.48 ± 6.47 ppm of hydroxybenzoic acid, 13.81 ± 0.85 ppm of vanillic acid, 12.48 ± 0.66 ppm of vanillin, 12.42 ± 0.87 ppm of* p*-coumaric acid, and 21.39 ± 1.45 ppm of ferulic acid were produced from lignin after MnP catalysis. Based on the results obtained, the yield of hydroxybenzoic acid was the highest, followed by ferulic acid and lastly vanillic acid, vanillin, and* p*-coumaric acid. However, the yields and recoveries of these compounds, except vanillic acid from MnP-catalyzed reaction, were found to be not significantly different (*p* > 0.05) with the experiment control. The yield of hydroxybenzoic acid, vanillin,* p*-coumaric acid, and ferulic acid from experiment control (reaction without MnP) were 468.58 ± 16.26, 11.74 ± 0.53, 12.30 ± 0.40, and 19.61 ± 0.58 ppm, respectively. However, the yield of vanillic acid was improved from 10.66 ± 0.81 to 13.81 ± 0.85 ppm in the presence of MnP. Thus, presumably, the release of these compounds was probably due to the oxidation reaction activated by other components in the reaction medium, but not due to the catalytic action of MnP. With this, the role of MnP in the catalysis of lignin deconstruction was found to be insignificant in this study. Such finding can be explained by the study [47] where the role of chelated Mn^3+^ ion in the oxidation of lignin/lignin model compounds was emphasized in their study. A prominent lignin oxidation reaction was found to occur with the presence of Mn^3+^ and chelator (such as oxalate, pyrophosphate) without MnP. In spite of that, lower rate of oxidation was also found to take place in the media containing Mn^2+^, chelator, and H_2_O_2_ only. Mechanism of MnP in oxidative catalysis was demonstrated by study [15, 23]. Catalytic cycle of MnP is initiated by the binding of H_2_O_2_ to ferric ion of MnP, forming iron-peroxide complex. Subsequently, MnP compound I (Fe^4+^-oxo-porphyrin-radical complex) formed, after the cleavage of peroxide oxygen-oxygen bond by accepting 2 electrons from heme of MnP. A monochelated Mn^2+^ ion will then act as an electron donor to the reduced MnP compound I into MnP compound II (Fe^4+^-oxo-porphyrin complex) by cleaving the dioxygen bond hydrolytically. As a result, a water molecule was generated and Mn^2+^ was oxidized to Mn^3+^. Similar mechanism was indicated for reduced MnP compound II to generate native MnP. Thus, for each MnP catalytic cycle, two Mn^3+^ ions and two water molecules were generated. During lignin degradation, reactive phenolic radical was formed by the nonspecific Mn^3+^ ion attack via hydrogen and one electron abstraction. The generated reactive radicals led to depolymerization of lignin. Besides, reaction system of MnP, chelator, Mn^2+^, and H_2_O_2_ was also found to catalyze C_*α*_-C_*β*_ cleavage, C_*α*_-oxidation, and alkyl-aryl cleavages of phenolic syringyl type *β*-1 lignin structure. However, only small part of aromatic structures of lignin comprised of phenolic hydroxyl groups. The abundant nonphenolic substructures of lignin are not susceptible to the attack by chelated Mn^3+^ ion [15, 48]. Moreover, undistinguished role of MnP in lignin degradation was also published in study [49]. In the study, meaningful lignin degradation cannot be achieved by single enzyme MnP, because the enzyme was only active in the initial stage of lignin depolymerization. Bonds cleavages near the end units of lignin polymer, where the free phenolic hydroxyl content is the highest, were predominant during MnP catalysis. With this, free phenolic compounds from the lignin network released. Such published findings supported our results in this study. Referring to the results in Table 2, the generated aromatic compounds were the esterified hydroxycinnamic acids originally present in the lignin network. Nonetheless, vanillin and vanillic acid were regarded as the oxidation products from free ferulic acid in the medium. Besides, oxidation of* p*-coumaric acid can also contribute to the yield of hydroxybenzoic acid.

### 3.5. Comparative Efficacy of Various Lignin Oxidation Methods

Based on previous results and discussion, it was found that the types and yields of compounds produced from lignin oxidation produced by various methods were different.  Table 3 showed the different types and yields of aromatic compounds obtained from nitrobenzene, direct oxygen, cutinase, and MnP oxidation of lignin. Generally, performance of enzymatic reaction (cutinase and MnP) was found to be poorer than chemical process (nitrobenzene and oxygen). For chemical oxidation, the rate of retroaldol reaction determined the efficacy of the reactions. In order to achieve highly effective oxidation, a more severe reaction environment such as high temperature, high pH, and strong oxidant has to be established. A wide variety of main products and by-products can generate, depending on the reaction condition [34, 37, 38]. Yet, specific bond cleavage, such as ester bond by cutinase, contributed to the efficiency of enzymatic reaction. The total of productive binding of active site to substrate determines the effectiveness of enzymatic conversion. In general, milder reaction condition was normally designed for enzymatic process. Physicochemical properties of large molecular weight substrate such as lignin may also affect the efficiency of active site binding and thus influence the overall performance of enzymatic reaction [42, 48, 50, 51]. In addition, the results also showed that enzyme cutinase was more efficient than MnP, in the release of aromatic compounds from lignin. The yields of aromatic compounds obtained from cutinase catalysis were higher than those obtained via MnP catalysis. As discussed in Section 3.4, it was proposed that the enzyme MnP has not been found to participate actively in oxidizing OPEFBF lignin. Study [49] suggested that lignin oxidation only can occur effectively in the presence of mixed enzyme MnP and lignin peroxidase. The products produced were solely caused by the action of Mn^3+^ ion generated via autooxidation between H_2_O_2_ and MnSO_4_. Restriction of Mn^3+^ to attack lignin is the main reason of insignificance of MnP in this catalysis. Furthermore, the success of monomeric aromatic compounds production through lignin hydrolysis by cutinase is a pioneering discovery in this study. Further study in this research is necessary for the improvement of the products' yield.

Overall, nitrobenzene oxidation was proved to be the best method of lignin fragment oxidation, followed by direct oxygen oxidation in generating aromatic compounds from lignin. This finding was supported by study [16], where nitrobenzene oxidation has been declared as the best oxidation method to produce high yield of aromatic aldehyde from lignin. However, study [27] reported an opposite finding. Direct oxygen oxidation has been suggested to be capable of producing higher yield of monomeric aromatic compounds than nitrobenzene oxidation when elevated temperature and pressure applied. Based on the data obtained, the yields of vanillin, syringic acid, and syringaldehyde obtained from nitrobenzene oxidation were the highest, while direct oxygen oxidation produced the highest yield of* p*-coumaric acid. This finding suggested that mild oxygen oxidation carried out in this study has preserved the* p*-coumaric acid from further oxidation. Besides, the highest yield of vanillin, syringic acid, and syringaldehyde from nitrobenzene oxidation also suggested that it was a more severe oxidation method than oxygen oxidation.

## 4. Conclusion

Among the tested protocols, nitrobenzene oxidation, followed by direct oxygen oxidation, was the best method in oxidizing lignin for the production of monomeric aromatic compounds. In this study, cutinase-catalyzed lignin hydrolysis was proposed for the first time in the production of hydroxybenzoic acid,* p*-coumaric acid, ferulic acid, syringaldehyde, and vanillin. Unexpectedly, cutinase-catalyzed lignin hydrolysis performed better than MnP-catalyzed lignin oxidation. Thus, further study on cutinase reaction on lignin is necessary.

## Figures and Tables

**Table:** (a) pH

Compounds	pH 7		pH 8		pH 10		pH 12	
	Yield	Recovery	Yield	Recovery	Yield	Recovery	Yield	Recovery
Hydroxybenzoic acid	153.14 ± 3.07^A^	7.66 ± 0.15^a^	165.30 ± 2.51^B^	8.27 ± 0.13^b^	162.72 ± 4.13^B^	8.14 ± 0.21^b^	156.14 ± 4.03^A^	7.81 ± 0.20^ab^
Syringaldehyde	13.89 ± 0.22^B^	0.70 ± 0.01^b^	14.94 ± 0.36^C^	0.75 ± 0.02^b^	13.93 ± 0.90^B^	0.70 ± 0.05^b^	2.71 ± 0.60^A^	0.14 ± 0.03^a^
Vanillin	3.33 ± 0.58^A^	0.17 ± 0.03^a^	8.02 ± 0.32^B^	0.40 ± 0.02^b^	8.49 ± 0.09^B^	0.42 ± 0.01^b^	9.55 ± 0.53^C^	0.48 ± 0.03^b^
*p*-Coumaric acid	7.04 ± 0.21^A^	0.35 ± 0.01^a^	7.72 ± 0.20^B^	0.39 ± 0.01^ab^	8.22 ± 1.18^B^	0.41 ± 0.06^b^	6.83 ± 0.45^A^	0.34 ± 0.02^a^
Ferulic acid	13.80 ± 0.63^B^	0.69 ± 0.03^b^	14.06 ± 0.13^B^	0.70 ± 0.07^b^	13.68 ± 0.38^B^	0.68 ± 0.02^b^	7.78 ± 0.39^A^	0.39 ± 0.02^a^

*Note*.

^A-B^Different alphabets in the same row indicated that there are significant differences (*p* < 0.05) between the yields from different pH.

^a-b^Different alphabets in the same row indicated that there are significant differences (*p* < 0.05) between the recoveries from different pH.

**Table:** (b) Lignin concentration

Compounds	2% wt.		5% wt.		8% wt.		10% wt.	
	Yield	Recovery	Yield	Recovery	Yield	Recovery	Yield	Recovery
Hydroxybenzoic acid	165.30 ± 2.51^A^	8.27 ± 0.13^b^	454.14 ± 11.66^B^	9.08 ± 0.23^c^	642.83 ± 14.45^C^	8.04 ± 0.18^b^	733.19 ± 26.68^D^	7.33 ± 0.27^a^
Syringaldehyde	14.94 ± 0.36^A^	0.75 ± 0.02^a^	49.70 ± 2.88^B^	0.99 ± 0.06^b^	70.19 ± 3.31^C^	0.88 ± 0.04^b^	77.78 ± 3.78^D^	0.79 ± 0.04^a^
Vanillin	8.02 ± 0.32^A^	0.40 ± 0.02^c^	16.65 ± 1.07^B^	0.33 ± 0.02^b^	22.80 ± 1.04^C^	0.29 ± 0.01^a^	25.39 ± 0.78^D^	0.25 ± 0.08^a^
*p*-Coumaric acid	7.72 ± 0.20^A^	0.39 ± 0.01^c^	19.07 ± 0.67^B^	0.38 ± 0.01^b^	27.06 ± 1.20^C^	0.34 ± 0.02^a^	29.13 ± 2.72^C^	0.29 ± 0.03^a^
Ferulic acid	14.06 ± 0.13^A^	0.70 ± 0.07^c^	39.02 ± 1.42^B^	0.78 ± 0.03^c^	50.19 ± 1.23^C^	0.63 ± 0.02^b^	56.95 ± 2.52^D^	0.57 ± 0.03^a^

*Note*.

^A-B^Different alphabets in the same row indicated that there are significant differences (*p* < 0.05) between the yields from different lignin concentration.

^a-b^Different alphabets in the same row indicated that there are significant differences (*p* < 0.05) between the recoveries from different lignin concentration.

**Table:** (c) Enzyme concentration

Compounds	0.625 mL cutinase g^−1^ lignin		1.25 mL cutinase g^−1^ lignin		1.875 mL cutinase g^−1^ lignin		2.5 mL cutinase g^−1^ lignin	
	Yield	Recovery	Yield	Recovery	Yield	Recovery	Yield	Recovery
Hydroxybenzoic acid	642.83 ± 14.45^B^	8.04 ± 0.18^b^	557.80 ± 12.76^A^	6.97 ± 0.16^a^	569.66 ± 11.48^A^	7.12 ± 0.14^a^	542.94 ± 27.88^A^	6.79 ± 0.35^a^
Syringaldehyde	70.19 ± 3.31^B^	0.88 ± 0.04^b^	53.94 ± 1.20^A^	0.67 ± 0.02^a^	54.09 ± 3.00^A^	0.68 ± 0.04^a^	54.94 ± 4.67^A^	0.69 ± 0.06^a^
Vanillin	22.80 ± 1.04^A^	0.29 ± 0.01^a^	21.68 ± 0.48^A^	0.27 ± 0.01^a^	21.87 ± 0.71^A^	0.27 ± 0.01^a^	20.77 ± 1.12^A^	0.26 ± 0.01^a^
*p*-Coumaric acid	27.06 ± 1.20^C^	0.34 ± 0.02^c^	22.81 ± 1.44^B^	0.29 ± 0.02^b^	21.78 ± 0.65^B^	0.27 ± 0.01^ab^	19.69 ± 0.25^A^	0.25 ± <0.00^a^
Ferulic acid	50.19 ± 1.23^B^	0.63 ± 0.02^b^	46.94 ± 1.59^A^	0.59 ± 0.02^a^	46.33 ± 3.56^A^	0.58 ± 0.04^a^	46.93 ± 2.81^A^	0.59 ± 0.04^a^

*Note*.

^A-B^Different alphabets in the same row indicated that there are significant differences (*p* < 0.05) between the yields from different enzyme concentration.

^a-b^Different alphabets in the same row indicated that there are significant differences (*p* < 0.05) between the recoveries from different enzyme concentration.

**Table:** (d) Time

Compounds	24 hours		48 hours		72 hours	
	Yield	Recovery	Yield	Recovery	Yield	Recovery
Hydroxybenzoic acid	642.83 ± 14.45^A^	8.04 ± 0.18^b^	592.59 ± 35.11^A^	7.41 ± 0.44^a^	649.62 ± 21.35^A^	8.12 ± 0.27^b^
Syringaldehyde	70.19 ± 3.31^C^	0.88 ± 0.04^c^	28.23 ± 0.55^A^	0.35 ± 0.01^a^	34.03 ± 2.01^B^	0.43 ± 0.03^b^
Vanillin	22.80 ± 1.04^A^	0.29 ± 0.01^a^	26.61 ± 1.80^B^	0.33 ± 0.02^b^	32.64 ± 2.35^C^	0.41 ± 0.03^c^
*p*-Coumaric acid	27.06 ± 1.20^B^	0.34 ± 0.02^b^	22.08 ± 0.59^A^	0.28 ± 0.01^a^	23.76 ± 1.63^A^	0.30 ± 0.02^ab^
Ferulic acid	50.19 ± 2.23^C^	0.63 ± 0.02^b^	41.77 ± 0.90^A^	0.52 ± 0.01^a^	43.39 ± 1.10^B^	0.54 ± 0.01^a^

*Note*.

^A-B^Different alphabets in the same row indicated that there are significant differences (*p* < 0.05) between the yields from different time.

^a-b^Different alphabets in the same row indicated that there are significant differences (*p* < 0.05) between the recoveries from different time.

**Table:** (e) Temperature

Compounds	40°C		55°C		65°C	
	Yield	Recovery	Yield	Recovery	Yield	Recovery
Hydroxybenzoic acid	492.54 ± 38.89^A^	6.16 ± 0.49^a^	557.80 ± 12.76^B^	6.97 ± 0.16^b^	498.88 ± 42.35^A^	6.24 ± 0.53^a^
Syringaldehyde	35.22 ± 2.01^A^	0.44 ± 0.03^a^	53.94 ± 1.20^B^	0.67 ± 0.02^b^	60.12 ± 4.26^C^	0.75 ± 0.05^c^
Vanillin	15.99 ± 0.74^A^	0.20 ± 0.01^a^	21.68 ± 0.50^B^	0.27 ± 0.01^b^	23.44 ± 2.21^B^	0.29 ± 0.03^b^
*p*-Coumaric acid	13.94 ± 1.29^A^	0.17 ± 0.02^a^	22.81 ± 1.44^B^	0.29 ± 0.02^b^	13.01 ± 2.15^A^	0.16 ± 0.03^a^
Ferulic acid	37.81 ± 2.35^B^	0.47 ± 0.03^a^	46.94 ± 1.59^C^	0.59 ± 0.02^b^	31.74 ± 2.80^A^	0.40 ± 0.04^a^

*Note*.

^A-B^Different alphabets in the same row indicated that there are significant differences (*p* < 0.05) between the yields from different temperature.

^a-b^Different alphabets in the same row indicated that there are significant differences (*p* < 0.05) between the recoveries from different temperature.

Yield of oxidation products was expressed in unit part per million (ppm), while recovery of oxidation products from lignin was expressed in unit milligram per gram of lignin (mg g^−1^ lignin).

**Table 2:** Oxidation products from the enzymatic catalysis of manganese peroxidase (MnP) (*p* < 0.05; *n* = 3).

Compounds	Without MnP		With MnP	
	Yield	Recovery	Yield	Recovery
Hydroxybenzoic acid	468.58 ± 16.26^A^	6.85 ± 0.24^a^	459.48 ± 6.47^A^	6.72 ± 0.10^a^
Vanillic acid	10.66 ± 0.81^A^	0.16 ± 0.01^a^	13.81 ± 0.85^B^	0.20 ± 0.01^b^
Vanillin	11.74 ± 0.53^A^	0.17 ± 0.01^a^	12.48 ± 0.66^A^	0.18 ± 0.01^a^
*p*-Coumaric acid	12.30 ± 0.40^A^	0.18 ± 0.01^a^	12.42 ± 0.87^A^	0.18 ± 0.01^a^
Ferulic acid	19.61 ± 0.58^A^	0.29 ± 0.01^a^	21.39 ± 1.45^A^	0.31 ± 0.02^b^

*Note*.

^A-B^Different alphabets in the same row indicated that there are significant differences (*p* < 0.05) between the yields from different catalysis.

^a-b^Different alphabets in the same row indicated that there are significant differences (*p* < 0.05) between the recoveries from different catalysis.

**Table 3:** Comparison of aromatic compounds yields from lignin oxidation/deconstruction through nitrobenzene oxidation, oxygen oxidation, cutinase catalysis and manganese peroxidase (MnP) catalysis (*n* = 3; *p* < 0.05).

Compounds	Compounds' recovery (mg g^−1^ lignin)			
	Nitrobenzene	Oxygen	Cutinase	MnP
Hydroxybenzoic acid	16.66 ± 2.47^c^	16.40 ± 1.98^c^	8.04 ± 0.18^b^	6.72 ± 0.10^a^
*p*-Hydroxybenzaldehyde	0.28 ± 0.01^a^	0.25 ± <0.00^a^	n.d	n.d
Vanillic acid	1.28 ± 0.08^b^	1.26 ± 0.06^b^	n.d	0.20 ± 0.01^a^
Vanillin	8.43 ± 1.16^d^	1.30 ± 0.11^c^	0.29 ± 0.01^b^	0.18 ± 0.01^a^
Syringic acid	3.77 ± 0.34^b^	2.08 ± 0.03^a^	n.d	n.d
Syringaldehyde	40.76 ± 2.09^c^	3.92 ± 0.04^b^	0.88 ± 0.04^a^	n.d
*p*-Coumaric acid	0.76 ± 0.11^c^	1.08 ± 0.06^d^	0.34 ± 0.02^b^	0.18 ± 0.01^a^
Ferulic acid	2.24 ± 0.17^c^	2.02 ± 0.13^c^	0.63 ± 0.02^b^	0.31 ± 0.02^a^

*Note*.

^a-b^Different alphabets in the same row indicated that there are significant differences (*p* < 0.05) between the mean values from different treatments.

n.d represents not detected.
